# The prevalence of symptomatic and asymptomatic malaria and its associated factors in Debre Elias district communities, Northwest Ethiopia

**DOI:** 10.1186/s12936-022-04194-7

**Published:** 2022-06-03

**Authors:** Abtie Abebaw, Yibeltal Aschale, Tadesse Kebede, Asrat Hailu

**Affiliations:** 1 Department of Medical Laboratory Science, College of Health Sciences, Debre Markos University, P.O. Box: 269, Debre Markos, Ethiopia; 2 Department of Microbiology, Immunology and Parasitology, College of Health Sciences, Addis Ababa University, Addis Ababa, Ethiopia

**Keywords:** Prevalence, Symptomatic malaria, Asymptomatic malaria, Debre Elias

## Abstract

**Background:**

Malaria is a major cause of morbidity and mortality worldwide. According to the World Health Organization 2021 malaria report, it is considered to be endemic in 85 countries and territories. Malaria elimination programmes have also faced many challenges, such as widespread asymptomatic carriers in endemic regions, and they should be considered in malaria-control programmes in endemic areas for successful transmission interruption. This study aimed to assess the prevalence of symptomatic and asymptomatic malaria infections, and associated factors in Debre Elias district communities, Northwest Ethiopia from May to Jun 2018.

**Methods:**

A community-based cross-sectional study was conducted among selected kebeles in Debre Elias district, Amhara region, North-western Ethiopia. Multi-stage sampling technique was carried out to select representative households. A total of 440 randomly selected households were included, of which one individual per household was sampled for laboratory examination. Malaria prevalence was determined by light microscopy of stained blood films and using CareStart™ Malaria HRP2/pLDH (Pf/Pv) Combo rapid diagnostic test (RDT). A structured questionnaire was employed to collect socio-demographic data and associated risk factors. Data entry and analysis were carried out using Epi data 3.1 and SPSS version 23 software, respectively. The association between dependent and independent variables was explored by using bivariate and multivariate logistic regression analyses. Statistically significant association was declared at *P*-value of < 0.05.

**Results:**

A total of 440 (333 asymptomatic and 107 symptomatic) individuals were included in this study. The overall prevalence of malaria was 5% with the majority (59.1%) of infections caused by *Plasmodium falciparum.* Among asymptomatic participants, 4.8% (n = 16, 95% CI = 2.6–7.3) and 4.2% (n = 14, 95% CI = 2.1–6.5) were diagnosed and confirmed by RDT and light microscopy respectively. Similarly, the prevalence of malaria among 107 symptomatic individuals was 7.5% (n = 8, 95% CI = 2.8–12.6) by either RDT or light microscopy. Utilization of insecticide-treated net (ITN), availability of ITN, house with eave, previous history of malaria infection, and family history of malaria infection were significantly associated with malaria infection (*P* < 0.05).

**Conclusion:**

In this study, the prevalence of asymptomatic and symptomatic malaria was moderate. Screening of both symptomatic and asymptomatic malaria in the community is very important to scale up intervention programmes.

**Supplementary Information:**

The online version contains supplementary material available at 10.1186/s12936-022-04194-7.

## Background

Malaria is a vector-borne life-threatening disease caused by protozoan parasites of the genus *Plasmodium* and transmitted by female *Anopheles* mosquitoes. There are four different human malaria species; namely *Plasmodium falciparum*, *Plasmodium vivax*, *Plasmodium malariae*, and *Plasmodium ovale*, but they differ in many aspects of their biology and geographic distribution. Among them, *Plasmodium falciparum* and *Plasmodium vivax* are the most prevalent [1]. A fifth species, *Plasmodium knowlesi*, which causes malaria in macaque monkeys has recently been reported to infect humans in Southeast Asia [2].

The clinical manifestation of *Plasmodium* infection varies from asymptomatic to severe and fatal malaria [3]. The disease may result in death or serious morbidity including impaired consciousness, respiratory distress, hypoglycaemia, and jaundice which result in admission to intensive care or prolonged hospital stay [1, 4]. Asymptomatic malaria infections likely serve as an important parasite reservoir by maintaining parasite transmission [5, 6]. Parasites from asymptomatic carriers are more infectious to the *Anopheles* mosquito [7] and a major source of gametocytes for local mosquito vectors, contributing to the persistence of malaria transmission [5].

In Ethiopia, malaria is one of the most common diseases which causes high morbidity and mortality [8]. It is prevalent in over 75% of the country’s area with altitudes below 2000 m, and 68% of the total population is at risk [8, 9]. *Plasmodium falciparum* and *Plasmodium vivax* are dominant *Plasmodium* species accounting for almost 60% and 40% of all cases, respectively. Whereas, *Plasmodium malariae* and *Plasmodium ovale* are rare and both account for less than 1% of all confirmed malaria cases. The major malaria vector incriminated in Ethiopia is *Anopheles arabiensis* followed by *Anopheles pharoensis*, *Anopheles funestus* and *Anopheles nili* [8, 10, 11]. Malaria mostly affects the population during planting and harvesting seasons when there is the greatest need for agricultural work. This leads to a heavy economic burden which can adversely affect the struggle against poverty [8, 9]. During epidemics, health facilities are overwhelmed with patients, and many resources are diverted to deal with the emergency [8]. A total of 1,848,231 cases and 173 deaths were reported globally due to malaria in 2020 [1].

Malaria control and elimination are becoming difficult in many countries including Ethiopia due to the presence of asymptomatic carriers, the emergence of insecticide resistance vectors and drug resistance *Plasmodium* species, and the lack of sustainable and predictable funds [1, 12]. Even if malaria prevalence declined globally, about 241 million malaria episodes (increases by 14 million from the previous year) and 627,000 deaths (increased by 12% from the previous year) occurred worldwide in 2020 with the vast majority of cases (95%) and 96% of malaria death occurring in Africa followed by South-East Asia. Most of this increase comes from countries in the World Health Organization (WHO) African Region [1].

Nonetheless, most studies on malaria focused on clinical manifestations, severity, and complication aiming to investigate and address the principal cause of malaria-related deaths. Data from health institutions represent a fraction of the entire infected population. Areas of high endemicity are at risk of asymptomatic malaria infection [13]. Asymptomatic malaria infections are typically detected through active surveillance [3]. In endemic areas, four to five asymptomatic carriers are found for every symptomatic malaria case [14]. Moreover, a small percentage of infected persons is sufficient to re-start malaria transmission in regions where there is a seasonal and low transmission of malaria [13, 15].

The national malaria strategic plan of Ethiopia aims to achieve nationwide malaria elimination by 2030 [9]. To achieve this goal, identifying both types of infections in the communities and treating them with appropriate anti-malarial drugs is very important for effective malaria control and prevention programmes [15–17]. Identifying symptomatic infections among different sex and age groups and promptly treating them with appropriate anti-malarial drugs is necessary for effective malaria control and prevention programmes [15–17]. However, for a longer-term and protracted impact, tackling the transmission of malaria from asymptomatic infections is essential. Asymptomatic malaria is recognized as an obstacle to malaria elimination. Despite some studies on asymptomatic malaria are done in different parts of Ethiopia but usually focus on children, pregnant women, and falciparum species only. Hence, knowledge of the current prevalence of symptomatic and asymptomatic malaria and its associated risk factors in the communities has paramount importance to scale up intervention programmes. There is no documented study done in Debre Elias district. Therefore, this study aimed to assess the prevalence of symptomatic and asymptomatic malaria and associated factors in Debre Elias district communities.

## Study area

The study was conducted in Debre Elias district, East Gojjam zone, Amhara region, North West Ethiopia. Debre Elias town is located 340 km northwest of Addis Ababa, the capital city of Ethiopia. Debre Elias is bordered in the South and West by Abay (Blue Nile) River which separates it from the Oromia region, in the Northwest by the West Gojjam zone, in the North by Machakel district and in the East by Gozamin district. The altitude of the district ranges from 800 to 2200 m above sea level and receives a mean annual rainfall of 1150 mmHg. The average daily temperature ranges from 18 to 27 °C. Based on the 2007 census result which is conducted by the Central Statistical Agency of Ethiopia, the district has a total population of 82,150. From this, 41,109 and 41,041 were men and women, respectively. Regarding geographical distribution, nearly one-tenth (9.65%) of the total population were urban inhabitants [18]. Debre Elias district has reported malaria every week and was identified as one of the malarious area by the East Gojjam zonal health office. The district has 15 rural *kebeles* (the lowest administrative unit in Ethiopia), of which seven are malarious.

## Study design and period

A community-based cross-sectional study was conducted from May to June 2018 among selected kebeles in Debre Elias district.

## Population

All family members living in the selected households and present during data collection were included in the study. Individuals who were taking anti-malarial therapy or who had been treated with anti-malarial drugs within the past 1 month before enrollment were excluded.

## Sample size determination

The sample size was determined by using a formula for a single population proportion. Since there was no study conducted in the area, a 14.5% prevalence was taken from a previous study conducted in Pawe, Benishangul-Gumuz Region, Ethiopia [19], with the margin of error (d) at 5%, and a design effect of 2 which gives a sample size of 382. By considering 15% non-response rate the total sample size becomes 440.

## Sampling techniques and procedure

A multi-stage sampling technique was used to select the representative sample size. Three out of seven malarious kebeles (Guay, Chago, and Genet) were selected randomly using a lottery method. To determine the proportional sample size for each selected kebele, the recent demographic registration of households available at the local administration was used. Based on the number of households, the estimated sample size was proportionally distributed to the selected three Kebeles; for Guay: 189, Chago: 93, and Genet: 158 households. Each household was selected using a systematic random sampling technique. Finally, one individual among the selected household members who were volunteers was selected randomly using a lottery method.

## Data collection and management

### Socio-demographic data collection

Socio-demographic and other associated factors (Additional file 1: Table S1) were collected by trained health professionals using a pre-tested structured questionnaire (modified from a malaria indicator survey household questionnaire [20]). The questionnaire was initially prepared in English and translated into Amharic language (national language). Body temperature and other clinical manifestations were examined by senior health officer to identify symptomatic and asymptomatic individuals. This examination was carried out after the selection of individuals from households.

### Blood sample collection

Blood samples were taken from each participant aseptically by finger prick using a disposable sterile lancet from each participant. The samples were then used for malaria examination using RDT (CareStart^TM^Malaria HRP2/pLDH (Pf/Pv) Combo) and to prepare thick and thin blood smears for microscopic examination (Giemsa staining).

### Blood film examinations

For each participant, thick and thin blood films were prepared directly from finger-prick blood on the same slide and labeled with a unique code. The thin blood smears were fixed with methanol for 30 s. Then, smears were stained with 3% Giemsa solution for 30 min after being air-dried. Following the standard protocols, the stained smears were examined with a light microscope by oil immersion (100×) objective to detect the presence of malaria parasites. The results were classified qualitatively as either negative (no malaria parasite seen), positive for specific *Plasmodium* species, or mixed infection. At least 100 high power fields (100× objective) were examined before reporting a negative result [21].

### CareStart^TM^Malaria HRP2/pLDH (Pf/Pv) Combo

This test was performed according to the manufacturer’s instructions (Access Bio, Inc., Addis Ababa, Ethiopia). The kit was labelled with the respective sample code and 5 μl of whole blood specimen was added to the sample well of the test device. Two drops of lysis buffer were added into the buffer well to lyse the cells, release the antigen and facilitate antibody recognition. The RDT test results were then read after 20 min and interpreted as negative or positive for *Plasmodium falciparum*, *Plasmodium vivax*, or mixed.

## Operational definitions

Symptomatic malaria: the presence of malaria related symptoms (fever i.e., axillary temperature ≥ 37.5 °C, chills, headache, vomiting, joint pain) within the past 2 days and at the time of examination and the presence of malaria parasites in blood [13, 22, 23].

Asymptomatic malaria: absence of malaria related symptoms within the past 2 days and at the time of the survey, and the presence of malaria parasites in blood [24, 25].

## Quality control

The quality of the questionnaire was assessed by conducting a pre-test before the data collection period. To ensure maximum participation, households with absentees at the first visit were revisited for the second time on the next day. All the test procedures and the interpretation of results were accomplished based on standard operating procedures (SOP).

## Data analysis and interpretation

Data were entered and cleaned using Epi-Data 3.1 version whereas the analysis was conducted by using the statistical package for social sciences (SPSS) version 23 software. Descriptive statistics were used to give a clear picture of dependent and independent variables. The frequency distribution of the variables was worked out and the association between dependent and independent variables was explored by using bivariate and multivariate logistic regression analysis. Those variables associated with the outcome variable in bivariate logistic regression analysis (*P*-value of < 0.25) were further subjected to multivariate analysis to control possible confounders. Statistically significant association was declared at *P*-value of < 0.05. Finally, the findings of the study were presented in text and table as appropriate.

## Results

### Socio-demographic characteristics

A total of 440 study participants were included in this study. Of this, more than half (54.3%) were females. The mean age of the participants was 25.98 (± 16.328 SD) years with an age range of 1–80 years. The 5–14 age group accounted for about a quarter (24.8%) of the participants. All of the study participants were Orthodox Christian followers. Of the total study participants, 82.2% (n = 362) were farmers, 37.5% (n = 165) were unable to read and write and 53.9% (n = 237) were single. About 264 (60%) of them have access to mass media. The average family size was 4.92 with a range of 1–9. The majority (55.9%) of individuals had a family size of 4–6 (Table 1).

**Table 1:** Socio-demographic characteristics of the participants, Debre Elias district; Northwest Ethiopia, May to Jun 2018 (n = 440)

Variables	Guay (n = 189)	Chago (n = 93)	Genet (n = 158)	Total number (%)
	Number (%)	Number (%)	Number (%)	
Sex				
Male	91 (48.1)	39 (41.9)	71 (44.9)	201 (45.7)
Female	98 (51.9)	54 (58.1)	87 (55.1)	239 (54.3)
Age group				
< 5	9 (4.8)	6 (6.5)	7 (4.4)	22 (5.0)
5–14	46 (24.3)	21 (22.6)	42 (26.6)	109 (24.8)
15–24	36 (19)	29 (31.2)	37 (23.4)	102 (23.2)
25–34	26 (13.8)	15 (16.1)	30 (19)	71 (16.1)
35–44	41 (21.7)	13 (14)	30 (19)	84 (19.1)
45–54	8 (4.2)	5 (5.4)	5 (3.2)	18 (4.1)
≥ 55	23 (12.2)	4 (4.3)	7 (4.4)	34 (7.7)
Marital status				
Single	92 (48.7)	57 (61.3)	88 (55.7)	237 (53.9)
Married	84 (44.4)	32 (34.4)	66 (41.8)	182 (41.4)
Divorced	13 (6.9)	0	4 (2.5)	17 (3.9)
Widowed	0	4 (4.3)	0	4 (0.9)
Educational status				
Cannot read and write	74 (39.1)	34 (36.6)	57 (36.1)	165 (37.5)
Read and write	57 (30.2)	34 (36.6)	47 (29.7)	138 (31.4)
1–8th grade	42 (22.2%)	18 (19.3)	32 (20.3)	92 (20.9)
≥ 9th grade	16 (8.5)	7 (7.50	22 (13.9)	45 (10.2)
Occupational				
Civil servant	7 (3.7)	2 (2.2)	4 (2.5)	13 (3.0)
Farmer	145 (76.7)	80 (86)	137(86.7)	362 (82.2)
Merchant	37 (19.6)	11 (11.8)	17 (10.8)	65 (14.8)
Family size				
≤ 3	51 (27)	24 (25.8)	35 (22.2)	110 (25.0)
4–6	102 (54)	52 (55.9)	92 (58.2)	246 (55.9)
≥ 7	36 (19)	17 (18.3)	31 (19.6)	84 (19.1)

### Symptomatic, asymptomatic, and overall malaria prevalence

From a total of 440 study participants, 333 (75.7%) were asymptomatic and the rest 107 (24.3%) were symptomatic. The overall prevalence of malaria diagnosed by microscopy and RDT was 5% (n = 22, 95% CI = 3–7) and 5.5%, (n = 24, 95% CI = 3.2–7.5), respectively. The prevalence of malaria among asymptomatic individuals was 4.8% (n = 16, 95% CI = 2.6–7.3) and 4.2%, (n = 14, 95% CI = 2.1–6.5) by RDT and light microscopy respectively. The prevalence of symptomatic malaria was 7.5%, (n = 8, 95% CI = 2.8–12.6) as diagnosed by either RDT or light microscopy (Table 2).

**Table 2:** Prevalence of asymptomatic and symptomatic malaria, Debre Elias district; Northwest Ethiopia, May to June 2018

Individuals	RDT		Microscopy		Total
	Pos, n (%)	Total	Pos, n (%)	Total	Pos, n (%)
Asymptomatic	16 (4.8)	333	14 (4.2)	333	16 (4.8%)
Symptomatic	8 (7.5)	107	8 (7.5)	107	8 (7.5%)
Overall	24 (5.5)	440	22 (5)	440	24 (5.5%)

The respective relative proportion of *Plasmodium falciparum* and *Plasmodium vivax* was 57.1%) (n = 8) and 42.9%) (n = 6) among parasitologically confirmed asymptomatic malaria cases. Among the 8 symptomatic individuals found in the survey, *Plasmodium falciparum* accounted for 62.5% (n = 5), and the rest three (37.5%) were due to *Plasmodium vivax* confirmed by either RDT or microscopy (Table 3).

**Table 3:** The relative proportion of *Plasmodium* species among confirmed cases by RDT and light microscopy, Debre Elias district; Northwest Ethiopia, May to June 2018

Malaria	RDT			Microscopy		
	Pf (%)	Pv (%)	Total Pos	Pf (%)	Pv (%)	Total Pos
Asymptomatic	10 (62.5)	6 (37.5)	16	8 (57.1)	6 (42.9)	14
Symptomatic	5 (62.5)	3 (37.5)	8	5 (62.5)	3 (37.5)	8
Overall	15 (62.5)	9 (37.5)	24	13 (59.1)	9 (40.9)	22

Pf: *P. falciparum*; Pv: *P. vivax*; Pos: positive; RDT: Rapid Diagnosis Test

### Malaria associated factors analysis

From a total of 440 households, 94.3% (415) owned at least one ITN (Additional file 1: Table S1). The average number of ITNs was 2.44 (± 0.805 SD) per household with a range of 1–9. One hundred thirty-one households had less than 0.5 ITN per individual and 284 had ≥ 0.5 ITN per individual. Out of the screened participants, 341 (82.2%) reported they had slept under ITNs daily, 42 (10.1%), occasionally and 32 (7.7%) did not sleep under ITNs. Indoor Residual Spraying (IRS) coverage and frequency were assessed during the survey, but none of the households were sprayed.

### Multivariate analysis of malaria with associated factors

Potential malaria associated factors that showed a P-value < 0.25 in the bivariate analysis were entered for multivariate analysis to control confounding factors. Twelve variables (sex, age group, occupation, family size, availability of ITN, ratio of ITN ownership per family size, ITN usage, presence of eave in the house, presence of any hole on the wall, outdoor activities at night, family history of malaria and previous malaria infection) were selected and entered into the backward stepwise multivariate logistic regression model (Additional file 1: Table S1). Four variables (ITN utilization, presence of eave in the house, previous history of malaria infection, and family history of malaria infection) were significantly associated with malaria infection (P < 0.05).

Individuals who did not use ITN were about 5.5 times more likely to be infected with malaria than those who used it daily [AOR = 5.47 (95% CI = 1.04–28.50)]. Individuals who lived in a house having an eave were about 3.4 times more likely to be infected with malaria than those who lived in a house without an eave [AOR = 3.35 (95% CI = 1.02–10.93)]. However, individuals who did not have previous malaria history were 0.89 times less likely to be infected with malaria than those who had a malaria infection history [AOR = 0.11 (95% CI = 0.02–0.58)]. Moreover, individuals who had a family history of malaria infection were about 3.9 times more likely to be infected with malaria than those who did not [AOR = 3.87 (95% CI = 1.10–13.61)] (Table 4).

**Table 4:** Bivariate and multivariate logistic regression analysis of associated factors for malaria, Debre Elias district; Northwest Ethiopia May to June 2018

Variables	Malaria		Bivariate analysis		Multivariate analysis	
	Pos	Neg	COR (95%CI)	*P* value	AOR (95%CI)	*P* value
Utilization of ITN						
Daily	7	334	1	1	1	1
Occasionally	5	37	6.44 (1.94–21.34)	0.002	4.51 (1.21–16.79)	0.025
Not using	3	29	4.93 (1.21–20.11)	0.026	5.47 (1.04–28.50)	0.044
Presence of eave in the house						
Yes	10	65	3.85 (1.64–9.05)	0.002	3.35 (1.02–10.93)	0.045
No	14	351	1	1	1	1
Previous malaria infection						
Yes	22	139	1	1	1	1
No	2	277	0.05 (0.01–0.19)	P < 0.001	0.11 (0.02–0.58)	0.01
Family history of malaria infection						
Yes	17	98	7.88 (3.17–19.55)	P < 0.001	3.87 (1.10–13.61)	0.035
No	7	318	1	1	1	1

*Pos:* positive, *Neg:* negative, *COR:* crude odds ratio, *AOR:* adjusted odds ratio, *CI:* confidence interval

## Discussion

### Prevalence of malaria (symptomatic and asymptomatic malaria combined)

This study shows that the overall prevalence estimate of malaria infection in the study area was 5% (95% CI = 3–7) which is comparable to a study conducted on several regions of Ethiopia (4.1%) [26] and the finding from Benna Tsemay district, Southwest Ethiopia (6.1%) [27]. However, higher prevalence rates have been reported from different parts of Ethiopia; such as in Dilla town and the surrounding rural areas, Southern Ethiopia (16%) [28], and East Shewa Zone, Central Ethiopia (25%) [29]. The possible reason for the discrepancy might be due to difference in the study period because the two above-mentioned studies [26, 29] were conducted during the major malaria transmission period whereas the present study was conducted during a minor malaria transmission period which might underestimate the prevalence. In other countries, malaria prevalence rates are even higher, e.g., 41.6% in Nigeria [30]; 36.3% in Tanzania [31], and 27.6% in India [15]. This difference might be due to the difference in the prevalence and burden of malaria in countries. Compared to other endemic countries in sub-Saharan Africa, malaria prevalence in Ethiopia is relatively low. But, countries like Nigeria and Tanzania are among the top countries with the highest number of malaria cases and deaths [1]. In addition, the difference might be due to the difference in the quality of houses, the nature of the study population (i.e. the study conducted in Nigeria and Tanzania was among pregnant women and children, respectively, which are high risk groups due to decline in immunity), sample size and study period. Moreover, the prevalence in this study sites was higher than the reports of studies conducted in Butajira, central Ethiopia (prevalence of 0.92%) [32]. The MIS reports of Ethiopia in 2007, 2011, and 2015 showed a malaria prevalence of 0.5–1.3% [20, 33, 34]. The possible reason for the difference could be the difference in geographical location (rainfall, temperature and altitude), sample size (i.e. the study conducted in Butajira and the MIS reports of Ethiopia were conducted on a larger sample size than the present study), the locally implemented malaria control programmes (for instance IRS was not applied during the study period). In addition, the MIS reports of Ethiopia cover the whole parts of the country (malarious area and non-malarious area) whereas, the present study was done relatively on the malarious area.

### Prevalence of asymptomatic malaria infection

The present study showed that the prevalence of asymptomatic malaria in Debre Elias district was 4.2% (95% CI = 2.1–6.5). This finding is comparable with the study conducted in Jiga (2.8%) [35] and Shalla district (5%) [36]. It is also comparable with a prevalence of 5% on Thailand–Myanmar border [37]. However, prevalence rates higher than the present study have been reported in other localities within Ethiopia; for instance, 14.5% in Pawe (western Ethiopia) [19], 6.8% in Sanja (northwest Ethiopia) [24], and 9.1% in Arbaminch (southwest Ethiopia) [25]. A higher prevalence of asymptomatic malaria was also reported in other countries, e.g., 77.6% in Nigeria [38], and 8% in Tanzania [31]. This difference might be attributed to differences in geographical location relative to the present study area as the above-mentioned area is highly malarious and the population is continuously exposed to malaria this might lead to the development of immunity which results in asymptomatic malaria [3]. Another possible reason might be the difference in study design, quality of houses, nature of population, sample size, and study period (due to the seasonality of malaria in Ethiopia). Malaria control and prevention strategies applied in the area also might be a possible reason for the difference. For instance, the ITN ownership of the present study was higher than the study conducted in Arbaminch. It is a fact that ITN is a very important tool for the prevention of malaria transmission from mosquito to human or vice versa [39]. The study conducted in Mirab Abaya district near Arbaminch town in southwest Ethiopia, which is known to be highly malarious reported only a 1.2% prevalence of asymptomatic malaria [16]. The reason for the difference might be differences in study population nature as the study conducted in Mirab Abaya was conducted among children only. Usually children develop severe malaria due to lower immune system. In addition, the reason for the discrepancy might be because of differences in sample size, and diagnostic technique. Moreover, the finding of the present study was higher than the studies conducted in China–Myanmar Border [17] and in Myanmar [40] the prevalence of asymptomatic malaria was reported as 0.3% and 1.44%, respectively. The possible reason for this discrepancy might be due to difference in prevalence of malaria in the countries according to the WHO 2021 report countries like China are near to malaria elimination phase [1], sample size, study design, and economic status (i.e. lack of constant budget is one of the challenges for the prevention of malaria).

### Prevalence of symptomatic malaria infection

This study showed that the prevalence of symptomatic malaria in Debre Elias district was 7.5% (95% CI = 2.8–12.6). This finding is similar to the prevalence (7.52%) reported for Kombolcha (northeast Ethiopia) [41]. In Ethiopia, a much higher prevalence was reported in the East Shewa zone, central Ethiopia (20.5%) [42] in Hadiya zone, Sothern Ethiopia (25.8%) [43], and in North-west Ethiopia (20.8%) [44]. This difference might be due to variation in study design and setting i.e. the study conducted in East Shewa, Hadiya zone, and Northern Ethiopia were carried out in health facilities which might overestimate the prevalence than a community-based study. A study conducted in the East Shewa zone was conducted during the major malaria transmission season and among children, whereas the present study was conducted during the minor malaria transmission season. In addition, lower ITN ownership (51%) reported from the study conducted in East Shewa Zone than in the present study might be the reason for the discrepancy. Moreover, outside Ethiopia, a lot higher prevalence rates have been reported, e.g. 64.5% in Tanzania [31] and 32.4% in China–Myanmar [17]. The reason for this discrepancy might be due to differences in nature of the population such as the study conducted in Tanzania was done among children who are a high-risk group to develop symptomatic malaria, epidemiology of malaria in the countries, and sample size (i.e. a study conducted in China–Myanmar was conducted among 34 suspected individuals this might lead to overestimating the prevalence).

Generally, in this study the prevalence of malaria among symptomatic and asymptomatic individuals was different. This difference may be due to that we have used different sample sizes and the diagnosis was done by low sensitive techniques, especially for the diagnosis of asymptomatic malaria [45]. Asymptomatic malaria infected individuals have developed partial immunity which clears the parasite and leads to low parasite density relative to symptomatic individuals. Due to this, the detection of asymptomatic malaria may be missed by low sensitivity techniques like Microscopy and RDT [31, 46].

### The relative proportion of *Plasmodium* species

In this study, *Plasmodium falciparum* was more predominant than *Plasmodium vivax* with a relative proportion of 59.1% and 40.1%, respectively. This finding is supported by a nationwide report in Ethiopia [20, 33, 34] and study reports from other parts of Ethiopia, such as Pawe [19], Sanja [24], and Mirab Abaya [16]. In contrast, *Plasmodium vivax* dominated over *Plasmodium falciparum* in studies conducted in Hadiya zone [43], East Shewa zone [29, 42], and in Jiga (Northwest Ethiopia) [35]. This difference might be due to variation in the epidemiological distribution of *Plasmodium* species in different parts of Ethiopia, and most likely due to altitudinal and climatic differences.

### Associated factors of malaria infections

In the present study, the status of malaria and bed net utilization were strongly associated. The odds of being infected with malaria were higher among individuals who did not use a bed net and those who used bed nets occasionally than those who used daily (p < 0.05). This finding is supported by a study conducted in Ethiopia [19, 24, 25, 43, 44] and Nigeria [30]. Insecticide-treated bed nets provide protection both to the individuals sleeping under them by deterring mosquito bites and family members by killing mosquitoes, thereby reducing the transmission of malaria parasites [39]. Ownership of ITN was 94.3%, though usage patterns differed among the study subjects. This finding is consistent with the study conducted in Jiga [35] and Benna Tsemay district (southwestern Ethiopia) [27]. Lower rates of ITN ownership have been reported by the studies carried out in Hadiya [43] and Arbaminch [25]. This difference might be due to the difference in the capacity of the health system, and the strengths of malaria control and prevention programmes.

Other associated factors which were identified as important determinants for malaria infection were house structure, previous history of malaria episodes, and family history of malaria. The finding showed that living in houses with eave have a higher risk of acquiring malaria infection than those in the houses without eave. This is supported by the study conducted in some localities within Ethiopia [47]. The presence of eave(s) might enable mosquitoes to enter inside houses, and this increases the probability of indoor mosquito bites.

Malaria infection was strongly associated with a previous history of malaria episodes as was also observed in Sanja (Northwest Ethiopia) [24]. In addition, the risk of malaria infection was higher in those individuals who had a family history of malaria. This might be due to family members with a history of malaria infection being a reservoir for *Plasmodium* parasites and the source of infection for the rest of the family members.

The qualitative study on the utilization of ITNs could have given more information on risk factors. The cross-sectional nature of the study, could not allow us to determine whether the exposure or the outcome occurred first. The study was conducted in one season, and so the period prevalence is expected to vary, due to the seasonally bimodal distribution of malaria in Ethiopia.

## Conclusion and recommendations

In this study, the overall prevalence estimate of symptomatic and asymptomatic malaria was moderate. Among *Plasmodium* species, *Plasmodium falciparum* is found to be the highest prevalent in the area. Insecticide-treated bed nets have been sufficiently distributed to the household; however, none of the houses were sprayed by IRS in a period of 1 year prior to the study period. Regarding associated factors, utilization of ITN, availability of ITN, house with eave, previous history of malaria infection, and family history of malaria infection were significantly associated with malaria infection.

The results of the study may also help the local health centers and concerned health offices to know the burden of malaria and prevalent species in the study area and to plan well-organized malaria prevention and control programmes and/or scale up the existing prevention and control mechanisms of malaria. Indicating which type of *Plasmodium* species are the most prevalent in the study area is essential for devising management strategies. In addition, it will help to evaluate the effectiveness of malaria interventions being implemented in the study area. Based on the findings, treating the malaria carriers especially asymptomatic ones during the minor transmission seasons is also very important to prevent reservoirs for the major transmission period. Furthermore, the study will be used as recent information for those who need to conduct further investigation in the area.

Further prevalence studies should be conducted by using highly sensitive and specific techniques such as PCR. During the screening of malaria in the community, both asymptomatic and symptomatic malaria should be considered in the implementation of the control/elimination programme such that the effectiveness of control strategies can be monitored by reliable metrics.

## Supplementary Information

**Additional file 1: Table S1.** Bivariate analysis of associated factors for malaria, Debre Elias district; Northwest Ethiopia, May to June 2018.

## Data Availability

The datasets used and/or analyzed during the current study are available from the corresponding author upon reasonable request.

## Abbreviations

IRS: Indoor Residual Spraying
ITNs: Insecticide-treated bed Nets
WHO: World Health Organization
RDT: Rapid Diagnostic Tests
MIS: Malaria indicator survey
