# Supplementary material for: The prevalence of symptomatic and asymptomatic malaria and its associated factors in Debre Elias district communities, Northwest Ethiopia
Source: Malar J. 2022 Jun 3;21:167. doi: 10.1186/s12936-022-04194-7 (PMC9166605; doi:10.1186/s12936-022-04194-7)
Supplement: Supplementary file 1 — Additional file 1: Table S1. Bivariate analysis of associated factors for malaria, Debre Elias district; Northwest Ethiopia, May to June 2018. [file 12936_2022_4194_MOESM1_ESM.docx]

**Additional file 1**

Table S1: Bivariate analysis of associated factors for malaria, Debre Elias district; Northwest Ethiopia, May to June 2018

| Variables | | Malaria | | Bivariate analysis (95% CI) | |
| --- | --- | --- | --- | --- | --- |
|  |  | Pos | Neg | COR (95%CI) | P value |
| Age | <5 | 0 | 22 | NA | 0.998 |
|  | 5-14 | 3 | 106 | 0.462 (0.09-2.37) | 0.355 |
|  | 15-24 | 6 | 96 | 1.02 (0.24-4.25) | 0.977 |
|  | 25-34 | 9 | 62 | 2.37 (0.60-9.23) | 0.213 |
|  | 35-44 | 3 | 81 | 0.60 (0.11-3.11) | 0.548 |
|  | >45 | 3 | 49 | 1 | 1 |
| Sex | Male | 16 | 185 | 2.49 (1.06-5.96) | 0.039* |
|  | Female | 8 | 231 | 1 | 1 |
| Availability of ITN | Yes | 15 | 400 | 1 | 1 |
|  | No | 9 | 16 | 15 (5.71-39.4) | P<0.001* |
| Utilization of ITN | Daily | 7 | 334 | 1 | 1 |
|  | Occasionally | 5 | 37 | 6.44 (1.94-21.34) | 0.002* |
|  | Not using | 3 | 29 | 4.93 (1.2-20.1) | 0.026* |
| Presence of Eave in the house | Yes | 10 | 65 | 3.85 (1.6-9.0) | 0.002* |
|  | No | 14 | 351 | 1 | 1 |
| Any Hole on the wall of house | Yes | 9 | 65 | 3.2 (1.3-7.7) | 0.008* |
|  | No | 15 | 351 | 1 | 1 |
| Presence of cattle on sleeping home | Yes | 1 | 6 | 2.97 (0.3-25.7) | 0.32 |
|  | No | 23 | 410 | 1 | 1 |
| Outdoor activities overnight | Yes | 7 | 57 | 2.5 (1.0-6.5) | 0.043* |
|  | No | 17 | 359 | 1 | 1 |
| Health education about malaria | Yes | 21 | 329 | 1 | 1 |
|  | No | 3 | 87 | 0.54 (0.15-1.8) | 0.328 |
| Previous malaria infection history | Yes | 22 | 139 | 1 | 1 |
|  | No | 2 | 277 | 0.046 (0.01-0.19) | P<0.001* |
| Family history of malaria | Yes | 17 | 98 | 7.88 (3.1-19.5) | P<0.001* |
|  | No | 7 | 318 | 1 | 1 |
| Distance of water bodies | <1000m | 15 | 234 | 1.2 (0.55-3.0) | 0.54 |
|  | >1000m | 9 | 182 | 1 | 1 |
| Ratio of ITN per family size | <0.5 | 9 | 122 | 3.4 (1.1-9.8) | 0.022* |
|  | >0.5 | 6 | 278 | 1 | 1 |
| Occupation | Civil servant | 0 | 13 | NA | 0.99 |
|  | Farmer | 23 | 339 | 4.3 (0.57-32.7) | 0.154 |
|  | Merchant | 1 | 64 | 1 | 1 |
| Formal Education | None | 19 | 284 | 2.9 (0.38-22.5) | 0.299 |
|  | Primary school | 4 | 88 | 2.0 (0.21-18.4) | 0.541 |
|  | ≥ grade 9^th^ | 1 | 44 | 1 | 1 |
| Family size | <3 | 4 | 106 | 1 | 1 |
|  | 4-6 | 10 | 236 | 1.12 (0.34-3.6) | 0.848 |
|  | >7 | 10 | 74 | 3.58 (1.08-11.8) | 0.037* |
| *P value less than 0.05, NA: Not Applicable, Pos: Positive, Neg: Negative, COR: Crude Odds Ratio, CI: Confidence Interval | | | | | |
